# Application of insulin signaling to predict insect growth rate in *Maruca vitrata* (Lepidoptera: Crambidae)

**DOI:** 10.1371/journal.pone.0204935

**Published:** 2018-10-04

**Authors:** Md. Abdullah Al Baki, Jin Kyo Jung, Rameswor Maharjan, Hwijong Yi, Jeong Joon Ahn, Xiaojun Gu, Yonggyun Kim

**Affiliations:** 1 Department of Plant Medicals, Andong National University, Andong, Korea; 2 Division of Crop Cultivation and Environment Research, Department of Central Area Crop Science, National Institute of Crop Science, Rural Development Administration, Suwon, Korea; 3 Department of Southern Area Crop Science, National Institute of Crop Science, Rural Development Administration, Miryang, Korea; 4 Research Institute of Climate Change and Agriculture, National Institute of Horticultural and Herbal Science, Rural Development Administration, Jeju, Korea; 5 College of Plant Protection, Fujian Agriculture and Forestry University, Fuzhou, Fujian Province, People’s Republic of China; Institute of Plant Physiology and Ecology Shanghai Institutes for Biological Sciences, CHINA

## Abstract

Insect growth is influenced by two major environmental factors: temperature and nutrient. These environmental factors are internally mediated by insulin/insulin-like growth factor signal (IIS) to coordinate tissue or organ growth. *Maruca vitrata*, a subtropical lepidopteran insect, migrates to different climate regions and feeds on various crops. The objective of this study was to determine molecular tools to predict growth rate of *M*. *vitrata* using IIS components. Four genes [insulin receptor (InR), Forkhead Box O (FOXO), Target of Rapamycin (TOR), and serine-threonine protein kinase (Akt)] were used to correlate their expression levels with larval growth rates under different environmental conditions. The functional association of IIS and larval growth was confirmed because RNA interference of these genes significantly decreased larval growth rate and pupal weight. Different rearing temperatures altered expression levels of these four IIS genes and changed their growth rate. Different nutrient conditions also significantly changed larval growth and altered expression levels of IIS components. Different local populations of *M*. *vitrata* exhibited significantly different larval growth rates under the same nutrient and temperature conditions along with different expression levels of IIS components. Under a constant temperature (25°C), larval growth rates showed significant correlations with IIS gene expression levels. Subsequent regression formulas of expression levels of four IIS components against larval growth rate were applied to predict growth patterns of *M*. *vitrata* larvae reared on different natural hosts and natural local populations reared on the same diet. All four formulas well predicted larval growth rates with some deviations. These results indicate that the IIS expression analysis explains the growth variation at the same temperature due to nutrient and genetic background.

## Introduction

Insect development is characterized by growth, molting, and metamorphosis [1]. Insects continuously grow in body weight under favorable environmental factors such as nutrients and warm temperature. However, their continuous growth periodically punctuates with molting to replace with new cuticle for subsequent increase of body size. Upon reaching critical body size, insects undergo metamorphosis of larva-to-pupa or nymph-to-adult [2]. Molting and metamorphosis are mediated by juvenile hormone and ecdysteroids [3]. In contrast, growth is mainly mediated by insulin-like peptides (ILPs) in insects [4].

Several ILPs have been identified in each insect species. The first ILP was serendipitously discovered during identification of prothoracicotropic hormone (PTTH) of *Bombyx mori* as a small PTTH. Later it was known to be bombyxin [5,6]. Through genome analysis, 38 bombyxins have been identified from *B*. *mori* [7]. Multiple ILPs are also found in *Acyrthosiphon pisum* (10 ILPs), *Anopheles gambiae* (8 ILPs), *Drosophila melanogaster* (7 ILPs), and *Tribolium castaneum* (4 ILPs) [8]. Like vertebrate ILPs, insect ILPs share a molecular domain structure containing signal peptide, A-chain, C-chain, and B-chain. After post-translational modification by proteolytic cleavage, a mature heterodimeric peptide consisting of A-B chains linked with disulfide bond is produced. These ILPs can mediate insect growth and reproduction, and control blood sugar level [9]. Insulin/insulin-like growth factor signal (IIS) pathway regulates nutrition-dependent growth rate, in which fat body is the central tissue that connects nutrients to ILP secretion from median neurosecretory cells (mNSCs) called ILP-producing cells (IPCs) in *Drosophila* [10]. Fat body can sense amino acids and send a nutritional signal called fat body-derived signal [11]. In response to high fat or high sugar diet, a fat body-derived leptin-like protein called Unpaired 2 is released to activate ILP secretion from IPC through GABAergic neurons [12]. In addition, glucose is directly sensed by fat body which produces a small peptide called CCHamide-2 to activate brain mNSCs to secrete ILPs [13].

ILPs use membrane receptor to transmit their signals into target cells. Only one type of insulin receptor (InR) has been identified in most known insects except *Apis mellifera* and *T*. *castaneum* that contain two isoforms [14,15]. Once ILP binds to InR, the downstream IIS is highly conserved among animals [9]. Briefly, ILP-bound receptor is activated by autophosphorylation which then phosphorylates insulin receptor substrate (INS) by receptor kinase activity. The phosphorylated INS recruits phosphoinositide-3-kinase to the membrane where it phosphorylates phosphatidylinositol-4,5- bisphosphate into phosphatidylinositol-3,4,5-trisphosphate (PIP3). Accumulated PIP3 levels then recruit phosphoinositide-dependent kinase (PDK) which activates serine-threonine protein kinase (Akt). Akt phosphorylates Forkhead Box O (FOXO) to prevent its translocation into nucleus while it phosphorylates tuberous sclerosis complex (TSC) to up-regulate protein translation by releasing eukaryotic initiation factor 4E (eIF4E) from its inhibitor 4E-binding protein (4EBP). Amino acid-mediated TOR (target of rapamycin) pathway can activate TSC and share its downstream signal with IIS.

*Maruca vitrata* (Lepidoptera: Crambidae), the legume pod borer, is an insect pest that damages leaves, flowers, or pods of leguminous crops in subtropical and tropical regions [16]. With climate change, this species migrates to northern temperate regions including Korea. First occurrence of *M*. *vitrata* in Korea was reported in 2004 in the adzuki bean (*Vigna angularis*) field [17]. Although it remains unclear whether *M*. *vitrata* overwinters in Korea [18], the larvae can become cold-hardy through supercooling capacity and production of trehalose, a cryoprotectant [19]. Trehalose is a main hemolymph sugar of *M*. *vitrata*. Its titer is regulated by IIS in response to feeding rhythm [20]. In the study, RNA interference (RNAi) of IIS components of InR, Akt, TOR, or FOXO significantly altered trehalose level of *M*. *vitrata*.

The legume pod borer is polyphagous. It exhibits host-associated variation in development [21]. Its developmental difference is partially explained by post-transcriptional action of 13 micro RNAs against genes associated with cell signaling, metabolism, and metamorphosis [22]. Especially, its nutrient-based growth can be understood by IIS and its downstream physiological roles because nutrients are essential for all cell growth. A systemic and coordinated growth of cells in multicellular organisms requires an endocrine signal in which ILP plays a crucial role in insects [4].

This study tested a hypothesis that IIS could modulate larval growth of *M*. *vitrata*. To this end, expression levels of four IIS components (InR, Akt, TOR, and FOXO) were analyzed under different nutritional conditions. Subsequently, each gene RNAi against these IIS components was applied to determine their roles in mediating larval growth. Furthermore, this study developed a nutrient-based insect growth model by correlating expression levels of these IIS components and the larval growth rate.

## Materials and methods

### Insect rearing

Larvae of *M*. *vitrata* were collected from adzuki bean field in Suwon, Korea in 2004 and reared on an artificial diet [17] under laboratory conditions: 25 ± 1°C temperature, 16:8 h (L:D) photoperiod, and 60 ± 10% relative humidity. No specific permissions were required for this location because the field studies did not involve endangered or protected species. Five larval instars (L1-L5) were recognized by ecdysis. Adults were fed 10% sucrose solution.

### Mammalian insulin treatment

One day old fifth instar (L5D1) larvae were used because of big size for injection and relatively long period to discriminate the difference in growth rates among treatments. Mammalian insulin used commercial porcine insulin (Sigma-Aldrich Korea, Seoul, Korea). After solubilizing the insulin powder with 1 M HCl, different concentrations (0.01, 0.1, 1, 10 mg/mL) were prepared with 100 mM phosphate-buffered saline (PBS, pH 7.4). The insulin was injected to larvae using a microsyringe (Hamilton, Reno, Nevada, USA) in a volume of 1 μL.

### Effect of different rearing conditions on larval development

For analysis of different temperatures, newly molted L1 larvae were kept in four different temperatures (15°C, 20°C, 25°C, and 30°C) with artificial diet. For analysis of different nutrients, six different diet compositions were prepared by altering amounts of adzuki bean and cowpea powders (S1 Table) based on the standard artificial diet [17]. Newly molted L1 larvae were fed with six different nutrients. Control larvae were fed with the standard artificial diet.

Each treatment used 30 individuals. Larval period was measured from L1 to pupation. Developmental rate was calculated by taking the inverse of larval period. Body weights of newly molted pupae were measured.

### Effect of different natural hosts on larval development

Six natural hosts of *M*. *vitrata* consisted of three legume species with different varieties: *Vigna unguiculata* Jangchae variety, *Glycine max* Daewon variety, *G*. *max* Pungsannamul variety, *G*. *max* Cheongja-3ho variety, *G*. *max* Socheongja variety, and *V*. *angularis* Hongeon variety. Newly molted L1 larvae (< 12 h) were used for this experiment. Each host treatment used 30 larvae that were reared under laboratory conditions until pupation. Leaves of natural hosts were used as diets. They were changed every two days. Developmental rate and pupal weight were measured as described above.

### Effect of different genetic background on larval development

Different local populations (S4 Fig) of *M*. *vitrata* larvae were collected and reared in the laboratory with the standard artificial diet. After mating and oviposition, adults were used to extract genomic DNAs (gDNAs). Subsequent generation of local populations was used to analyze larval developmental rate. In each local population, 30 L1 larvae were reared under the same temperature and nutrient conditions (25°C and the standard artificial diet). They were used to measure larval periods and pupal weights.

### Random amplification of polymorphic DNA (RAPD) and genetic distance estimation

To extract gDNA, each individual was homogenized with a pestle in 500 μL of 20% Chelex 100 (Bio-Rad, Hercules, CA, USA) and heated at 100°C for 10 min. After cooling on ice for 2 min, the suspension was centrifuged at 10,000 x *g* for 3 min. The resulting supernatant was used as gDNA sample.

N-8041 RAPD primer (5'-ATCGGGTCCG-3') was used to discriminate populations of *M*. *vitrata*. PCR reaction mixture (25 μL) consisted of 1 μL of gDNA, 2.5 μL of 10x PCR buffer, 2.5 μL of dNTP, 2 μL of RAPD primer, 1 μL of Taq polymerase (GeneAll, Seoul, Korea), and 16 μL of deionized distilled water. Using MyCycler Personal Thermal Cycler (BioRad, Hercules, CA, USA), PCR was run with a preheating step at 94°C for 2 min followed by 35 cycles of denaturation at 94°C for 1 min, annealing at 48°C for 1 min, and extension at 72°C for 1 min. After 35 cycles, a polymerization reaction at 72°C for 10 min was added and the reaction was terminated by incubating at 4°C. PCR products were separated on 1% agarose gel in 1x TAE buffer under constant voltage (90 V) for 60 min. PCR bands were stained with ethidium bromide and observed under UV. For each population, 30 individuals were randomly chosen except for some populations with less than 30 samples (in which the total collection was used for analysis). Each separated PCR band was regarded as RAPD gene locus. Gene frequencies were calculated for each population. Genetic distance among populations was estimated by hierarchical cluster analysis using PROC CLUSTER program [23].

### Quantification of IIS gene expression levels

Reverse transcriptase-quantitative polymerase chain reaction (RT-qPCR) was used to determine IIS gene expression levels in L5 larvae on the first day (L5D1). Total RNAs were extracted from L5D1 larvae in all treatments in this study using Trizol reagent (Invitrogen, Carlsbad, CA, USA) according to the manufacturer’s instructions. After RNA extraction, RNA was resuspended in nuclease-free water and quantified using a NanoDrop spectrophotometer (Thermo Scientific, Wilmington, DE, USA). RNA (1 μg) was used for cDNA synthesis with RT PreMix (Intron Biotechnology, Seoul, Korea) containing oligo dT primer according to the manufacturer’s instructions.

Expression levels of four IIS genes were measured using a Real-Time PCR machine (Step One Plus Real-Time PCR System, Applied Biosystem, Singapore) under a guideline of Bustin et al. [24]. Real-time PCR was conducted in a 20 μL reaction volume containing 10 μL of Power SYBR Green PCR Master Mix (Thermo Scientific Korea, Seoul, Korea), 5 μL of cDNA template (50 ng), and 1 μL each of forward and reverse primers (S2 Table). After 10 min of an initial denaturation step, qPCR was performed with 40 cycles of denaturation at 95°C for 1 min, annealing at 52–58°C (S2 Table) for 30 s, and extension at 72°C for 40 s. The expression level of actin is relatively constant in different developmental stages or tissues [20]. Therefore, it was used as a reference gene to normalize target gene expression. Quantitative mRNA level of each gene in specific treatment was relatively estimated using the comparative CT (2^-ΔΔCT^) method [25] compared to gene expression level in the laboratory strain reared on the standard artificial diet at 25°C.

### RNA interference (RNAi) of IIS component gene expression

RNAi was performed using gene-specific dsRNA which was prepared with Megascript RNAi Kit (Ambion, Austin, TX, USA) according to the manufacturer’s instruction. Briefly, gene-specific primers were prepared by adding T7 sequence (5'-TAATACGACTCACTATAGGGAGA-3') at 5' end. According to PCR method described above, short PCR products (≈ 400 bp) were prepared using T7 sequence containing primers. Using T7 RNA polymerase, dsRNA was synthesized *in vitro* with NTP mixture at 37°C for 3 h. The resulting dsRNA was then mixed with Metafectene PRO (Biontex, Plannegg, Germany) at 1:1 ratio and incubated at 25°C for 20–30 min to form liposome. The dsRNA (1 μg) in liposome was injected to newly molted L5D1 larval hemocoel with a microsyringe (Hamilton, Reno, NV, USA). Treated larvae were fed with standard artificial diet. As dsRNA control (dsCON), dsRNA specific to CpBV-ORF302 was injected. Larval period was measured from L5D1 to pupation. Developmental rate was calculated by taking the inverse of larval period. Body weights of newly molted pupae were measured. Each treatment used 30 individuals.

### Statistical analysis

All studies were performed with three independent replications. Results are presented as mean ± standard deviation (SD) and plotted with SigmaPlot (Systat Software, San Jose, CA, USA). Means were compared with least square difference (LSD) test of one-way analysis of variance (ANOVA) using PROC GLM of SAS program [23] and discriminated at Type I error = 0.05.

## Results

### IIS facilitates larval growth of *M*. *vitrata*

**Fig 1:**
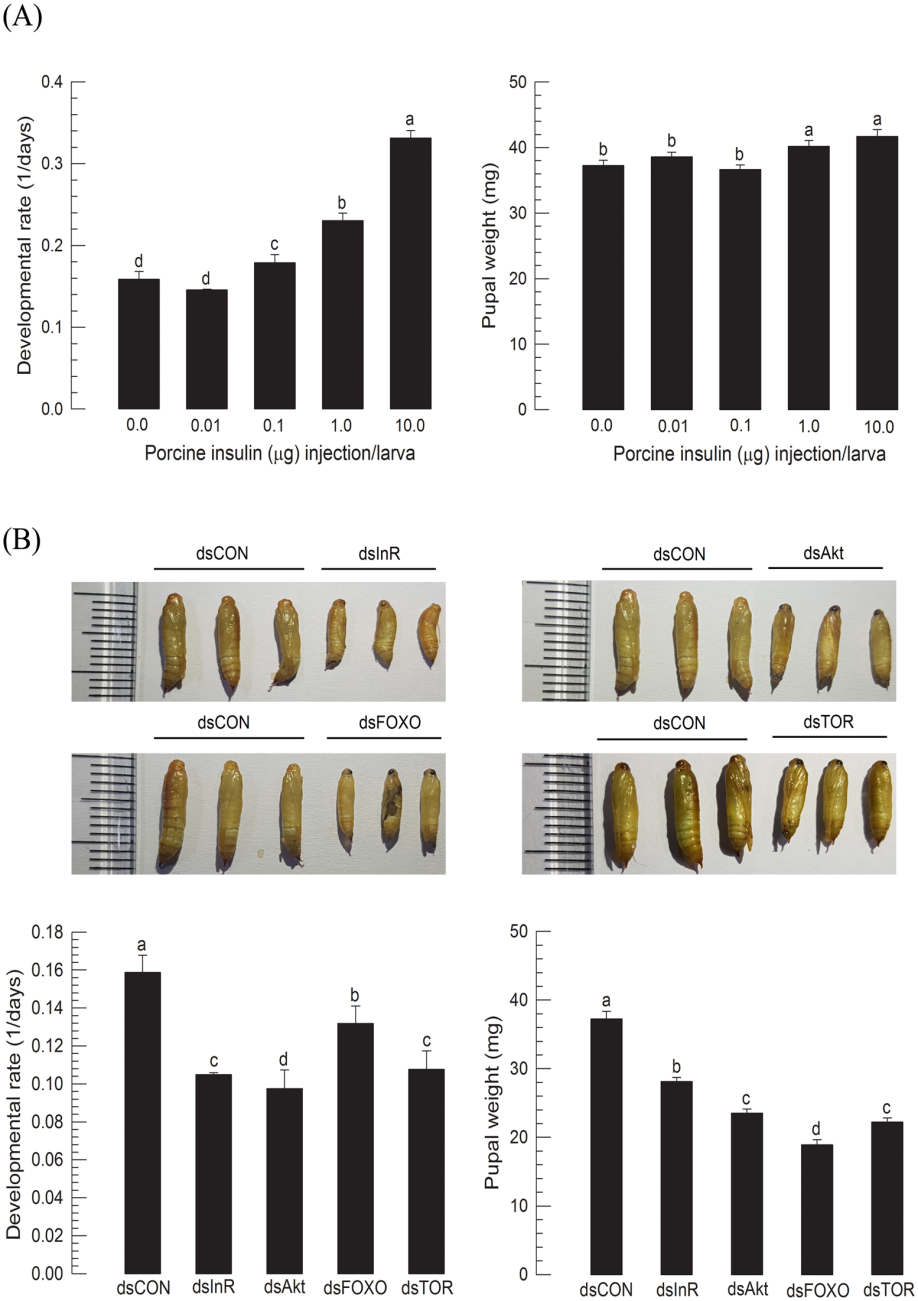
Effect of IIS components on larval growth of *M*. *vitrata*. (A) Stimulating effect of a porcine insulin on larval growth at 25°C rearing temperature. L5D1 (newly molted < 12 h) larvae were injected with different doses of insulin. Each dose was applied to 30 larvae. Developmental rate was calculated by inverse of the elapsed time (days) from injection to pupation. Pupal weight was measured within 8 h after pupation. (B) RNAi effect of IIS components (InR, Akt, FOXO or TOR) on larval growth. RNAi was performed by injecting 1 μg of gene-specific dsRNA to L5D1. A viral gene, CpBV302, was used as control dsRNA (‘dsCON’). Each RNAi treatment used 15 larvae. Different letters above standard deviation bars indicate significant difference among means at Type I error = 0.05 (LSD test).

Influence of IIS on larval growth of *M*. *vitrata* was tested by injection of porcine insulin. The mammalian insulin was effective to trigger IIS to modulate hemolymph trehalose levels [20]. Insulin facilitated larval growth in a dose-dependent manner by reducing larval period (Fig 1A). The insulin treatment also increased pupal weight of *M*. *vitrata*. This suggests that IIS mediates larval growth of *M*. *vitrata*.

Four IIS components (InR, Akt, FOXO, TOR) were analyzed for their domain composition and phylogenetic relationship with other orthologs of different insect species (S1 Fig). They shared high sequence homologies. For example, *M*. *vitrata* InR, Akt, FOXO, and TOR shared 87%, 76%, 83%, and 58% sequence homologies with the corresponding genes of *B*. *mori*, respectively. RNAi of these four IIS components interfered with larval growth of *M*. *vitrata* (Fig 1B). For all four IIS components, RNAi efficiencies were more than 60% for at least 2 days after dsRNA injection (S2 Fig). These RNAi treatments resulted in significant larval mortalities (43.3%). They also reduced pupal weights. Especially, RNAi specific to FOXO reduced almost 50% of pupal weight. RNAi treatments also significantly prolonged the larval period. These results indicated a functional association between IIS and larval growth in *M*. *vitrata*.

### Fluctuation of IIS component gene expression under different ambient temperatures

**Fig 2:**
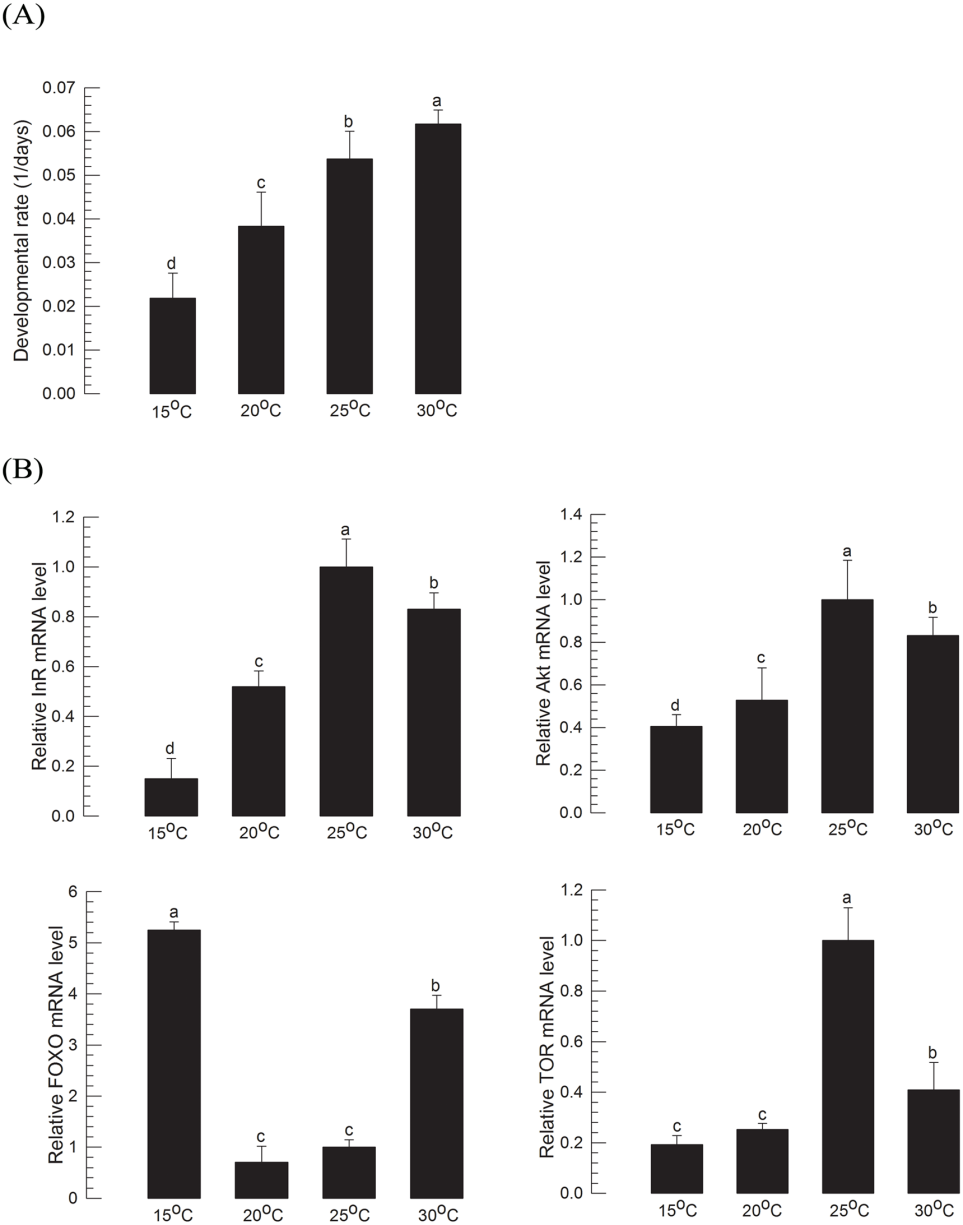
Effect of ambient temperature on larval growth of *M*. *vitrata*. (A) Change in developmental rates at four different temperatures. Newly hatched larvae were reared until pupation with an artificial diet. Developmental rate was calculated by inverse of the total larval period (days). Each temperature treatment used 30 larvae. (B) Change in expression levels of four IIS component genes (InR, Akt, FOXO, and TOR). These four genes’ expression levels in L5D1 larvae reared at different temperatures from L1 were quantified. Each treatment used nine randomly selected larvae. mRNA levels were relatively quantified and compared to mRNA levels in L5D1 reared at 25°C. Actin was used as reference gene for RT-qPCR to normalize target gene’s expression level. Different letters above standard deviation bars indicate significant difference among means at Type I error = 0.05 (LSD test).

Increase of ambient temperature from 15°C to 30°C facilitated larval development of *M*. *vitrata* (Fig 2A). However, expression levels of four IIS components did not exhibit high correlations with developmental rates (S3 Fig). Expression levels of InR and FOXO were higher at lower temperature while those of Akt and TOR were higher at higher temperatures (Fig 2B). However, these tendencies were not linearly dependent. To predict larval growth of *M*. *vitrata* using IIS component expression levels, a constant temperature condition may be required. This indicates that the prediction of larval growth rate using IIS may be applicable only in the same temperature.

### Nutritional factors influence IIS gene expression

**Fig 3:**
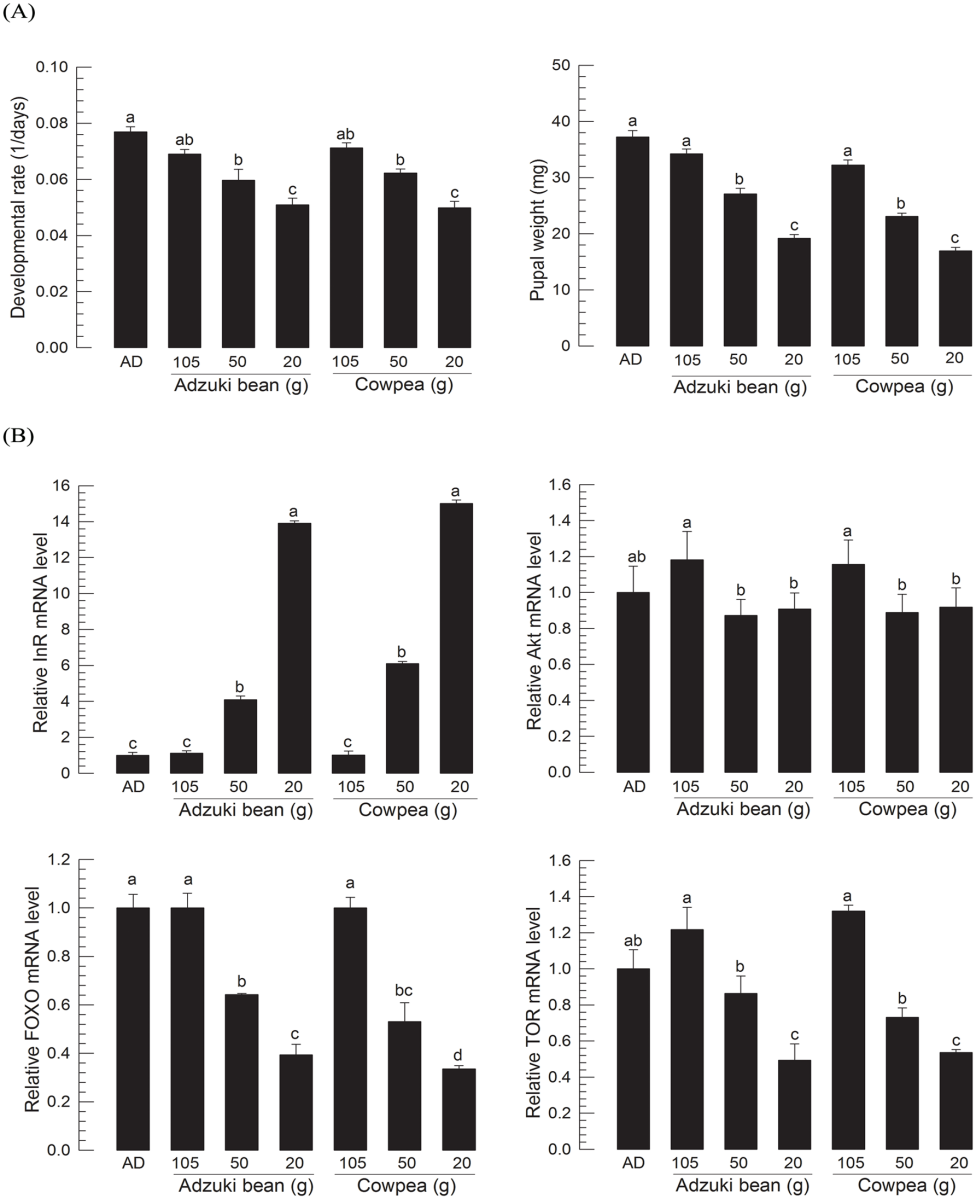
Effect of different nutritional diets on larval growth of *M*. *vitrata*. Newly hatched larvae (L1D1) were treated with seven different diets at a constant temperature of 25°C: a standard artificial diet (‘AD’), three adzuki bean diets at different nutritional amounts, and three cowpea diets at different nutritional amounts (S1 Table). (A) Diet effect on larval growth. Developmental rate was calculated by inverse of time period (days) from L1D1 to L5D1 (the first day of fifth instar). Pupal weight was measured within 8 h after pupation. Each diet treatment used 30 larvae. (B) Changes in expression levels of four IIS component genes (InR, Akt, FOXO, and TOR). These four genes’ expression levels in L5D1 of larvae reared with different diets from L1 were quantified. Each treatment used nine randomly selected larvae. mRNA levels were relatively quantified and compared to mRNA level of L5D1 reared with AD diet. Actin was used as reference gene in RT-qPCR to normalize target gene’s expression level. Different letters above standard deviation bars indicate significant difference among means at Type I error = 0.05 (LSD test). (C) Regression between gene expression levels and developmental rates. Dot lines indicate regression lines. Resulting regression equations are shown in Table 1.

Larval development of *M*. *vitrata* was modulated by nutrient amount (Fig 3) at 25°C rearing temperature. Artificial diets were prepared by using different amounts of two main diet components (adzuki bean and cowpea) and fed to larvae. Developmental rate and pupal weight were significantly (*P* < 0.05) different among different diets (Fig 3A) at a constant temperature of 25°C. Different diets also significantly (*P* < 0.05) changed gene expression levels of four IIS components (Fig 3B). Developmental rate exhibited a negative correlation with InR expression level, but positive correlations with gene expression levels of the other three components (Fig 3C). Based on these significant (*P* < 0.05) correlations, four IIS-growth regression models were estimated (Table 1).

**Table 1:** Prediction models of larval growth of *M*. *vitrata* using insulin/IGF signaling (IIS) at 25°C.

IIS components	Regression ^1^	R^2^	T test of slope		
			*t*	df	*P*
InR	Y = -0.0016 × X + 0.0726	0.8734	-20.60	62	3.66E-29
Akt	Y = 0.0381 × X + 0.0249	0.3506	5.74	62	3.2E-07
FOXO	Y = 0.0333 × X + 0.0337	0.5077	7.93	62	5.79E-11
TOR	Y = 0.0262 × X + 0.0394	0.7131	12.31	62	3.47E-18

^1^ For regression lines, ‘Y’ represents developmental rate (1/days) and ‘x’ represents relative expression level of mRNA compared to standard condition (25°C for a laboratory strain).

### Application of IIS-growth models under different natural hosts

**Fig 4:**
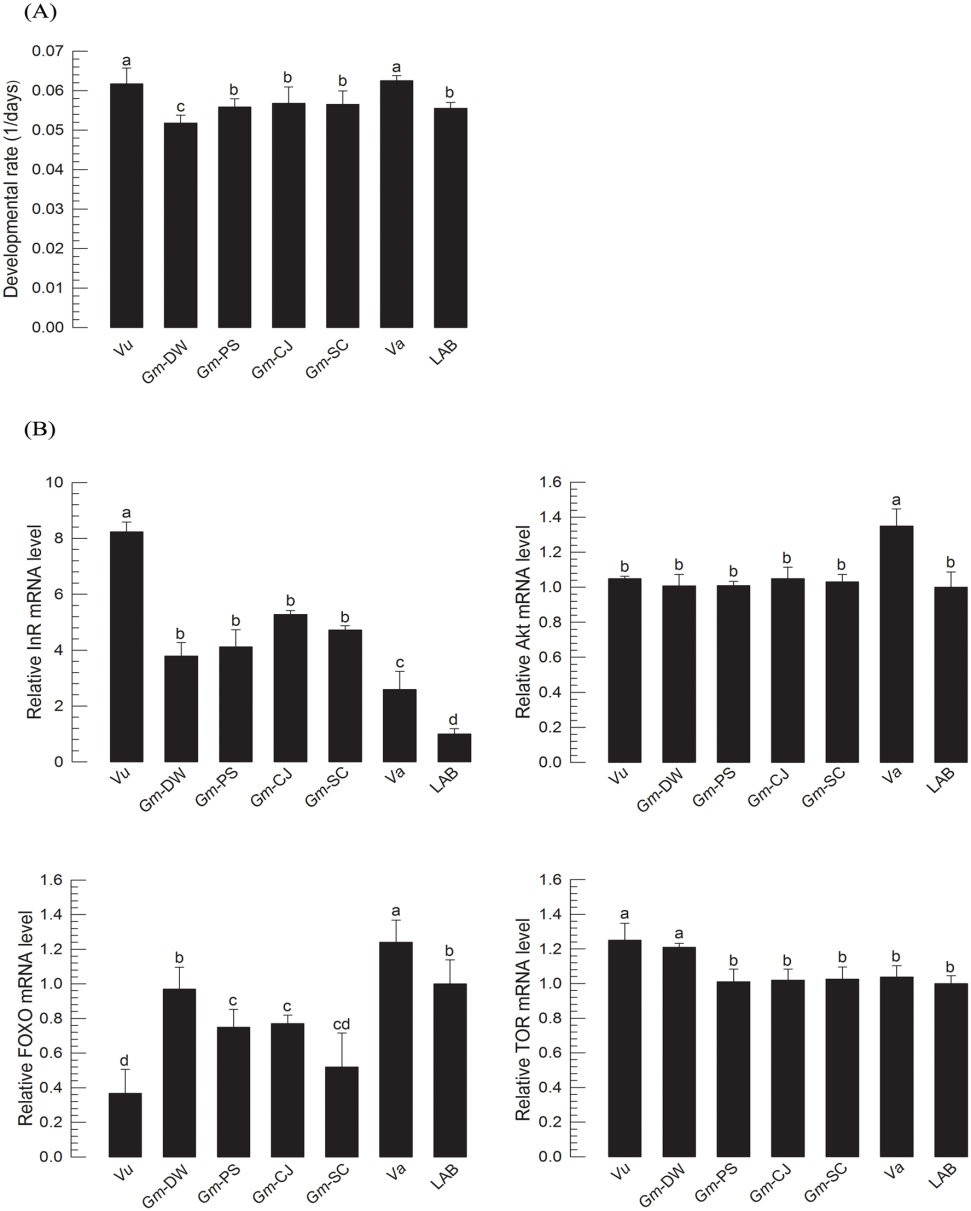
Validation of IIS-growth regression model between IIS expression levels and larval developmental rate of *M*. *vitrata*. Practical growth patterns of *M*. *vitrata* larvae were obtained by rearing larvae in the laboratory with artificial diet (‘LAB’) and six natural hosts: *Vigna unguiculata* Jangchae (‘Vu’), *Glycine max* Daewon (‘Gm-DW’), *Glycine max* Pungsannamul (‘Gm-PS’), *Glycine max* Cheongja-3ho (‘Gm-CJ’), *Glycine max* Socheongja (‘Gm-SC’), and *Vigna angularis* Hongeon (‘Va’). (A) Effect of host on larval growth. Developmental rate was calculated by inverse of the total larval period (days). Each host treatment used 30 larvae. (B) Change in expression levels of four IIS component genes (InR, Akt, FOXO, and TOR). These four genes’ expression levels in L5D1 of larvae reared on different hosts from L1 were quantified. Each treatment used nine randomly selected larvae. mRNA levels were relatively quantified and compared to mRNA levels in laboratory strain L5D1 reared with artificial diet. Actin was used as reference gene in RT-qPCR to normalize target gene’s expression level. Different letters above standard deviation bars indicate significant difference among means at Type I error = 0.05 (LSD test). (C) T-tests between the expected larval period (L1D1-L5D5) from regressions obtained from IIS gene expression levels and the observed larval period (L1D1-L5D5). Asterisk indicates significant difference between expected and observed values. ‘NS’ represents no significance at Type I error = 0.05.

Larvae of *M*. *vitrata* were reared on different natural hosts (Fig 4). Larval growth rates were compared to the reference growth rate of larvae reared with artificial diet in the laboratory at 25°C rearing temperature (Fig 4A). Different hosts significantly (*F* = 664.3; df = 6, 14; *P* < 0.0001) influenced larval developmental rate of *M*. *vitrata*. IIS gene expression also varied among different hosts (Fig 4B). Four models predicted the larval growth rates on Gm-CJ and Gm-SC hosts (Fig 4C). However, none of these models could effectively predict the growth rates on Gm-DW host. These results indicate the variation of larval growth rate of *M*. *vitrata* with different nutrient conditions under a constant temperature. Some of the nutritional variation of larval growth rate was explained and predictable by IIS component expression levels.

### Application of IIS-growth models under different genetic backgrounds

**Fig 5:**
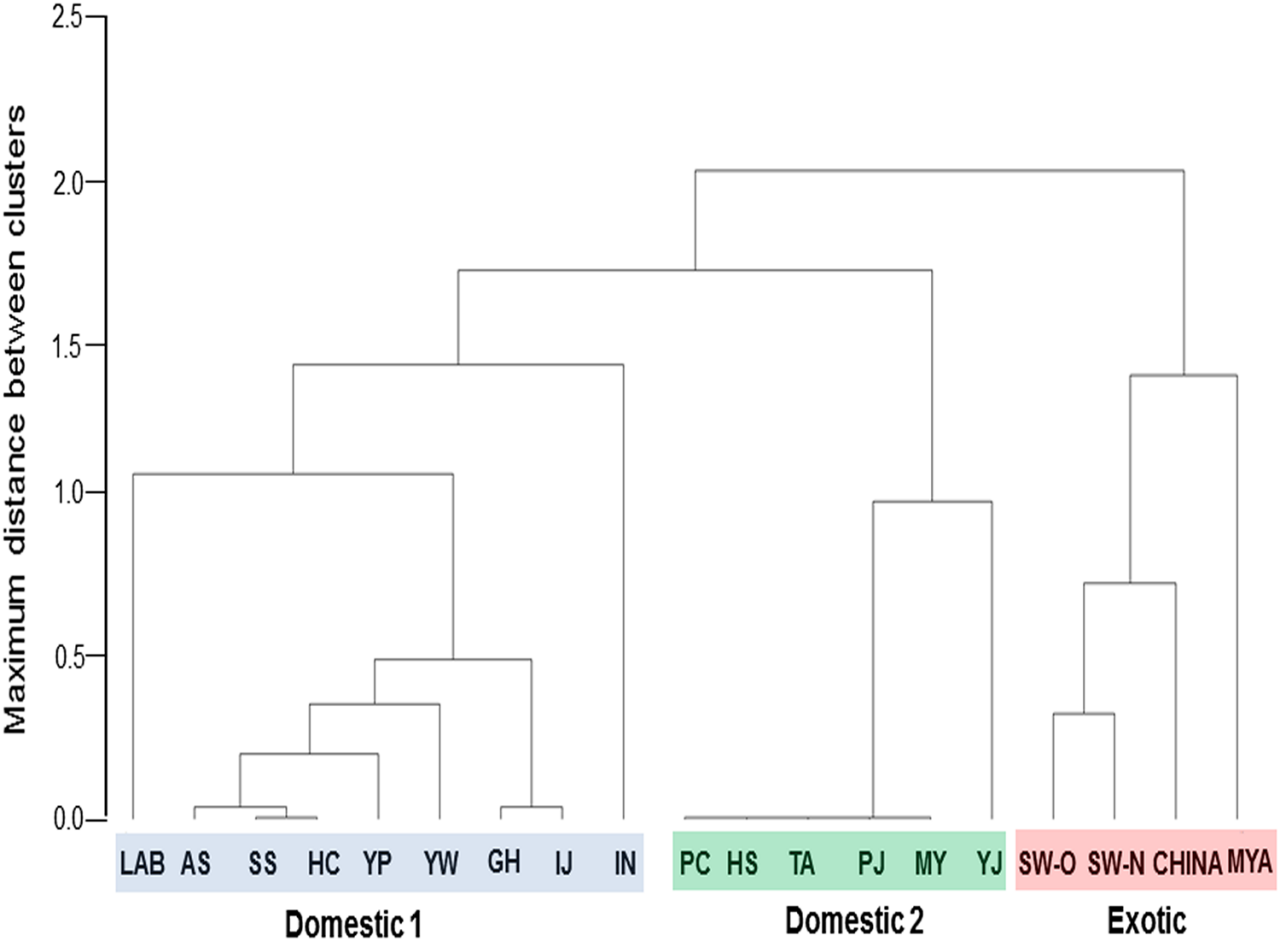
Genetic distance analysis among 19 local populations of *M*. *vitrata* using RAPD. Hierarchical clustering was performed using band polymorphism obtained from RAPD 8041 primer. These local populations included Myanmar (‘MYA’), China (‘CHINA’), Suwon 2018 (‘SW-N’), Suwon 2017 (‘SW-O’), Ganghwa (‘GH’), Pyungchang (‘PC’), Hoeungsung (‘HS’), Seosan (‘SS’), Ansan (‘AS’), Yeongju (‘YJ’), Paju (‘PJ’), Iksan (‘IS’), Miryang (‘MY’), Taean (‘TA’), Youngwol (‘YW’), Yangpyeong (‘YP’), Inje (‘IJ’), Hongchun (‘HC’), and a laboratory strain (‘LAB’). Local coordinates are 37°5′12.7″N 127°22′28.1″E for AS, 36°53′53.71″N 127°9′47.97″E for CA, 37°48′03.1″N 126°14′03.3″E for GH, 37°46′27″N 128°00′57″E for HC, 37°26′28″N 128°8′46″E for HS, 38°1′25″N 128°17′0″E for IJ, 35°44′29.04″N 129°2′14.28″E for KJ, 35°29′40.22″N 128°44′11.52″E for MY, 37°22′46.722″N 128°21′55.985″E for PC, 37°58′13.52″N 126°56′44.75″E for PJ, 36°45′57.22″N 126°30′11.64″E for SS, 37°15′43″N 126°59′15″E for SW, 36°40′6.47″N 126°18′4.95″E for TA, 37°6′56″N 127°23′11″E for YI, 37°33′16.81″N 127°43′03.19″E for YP, and 37°11′18″N 128°22′20″E for YW.

**Fig 6:**
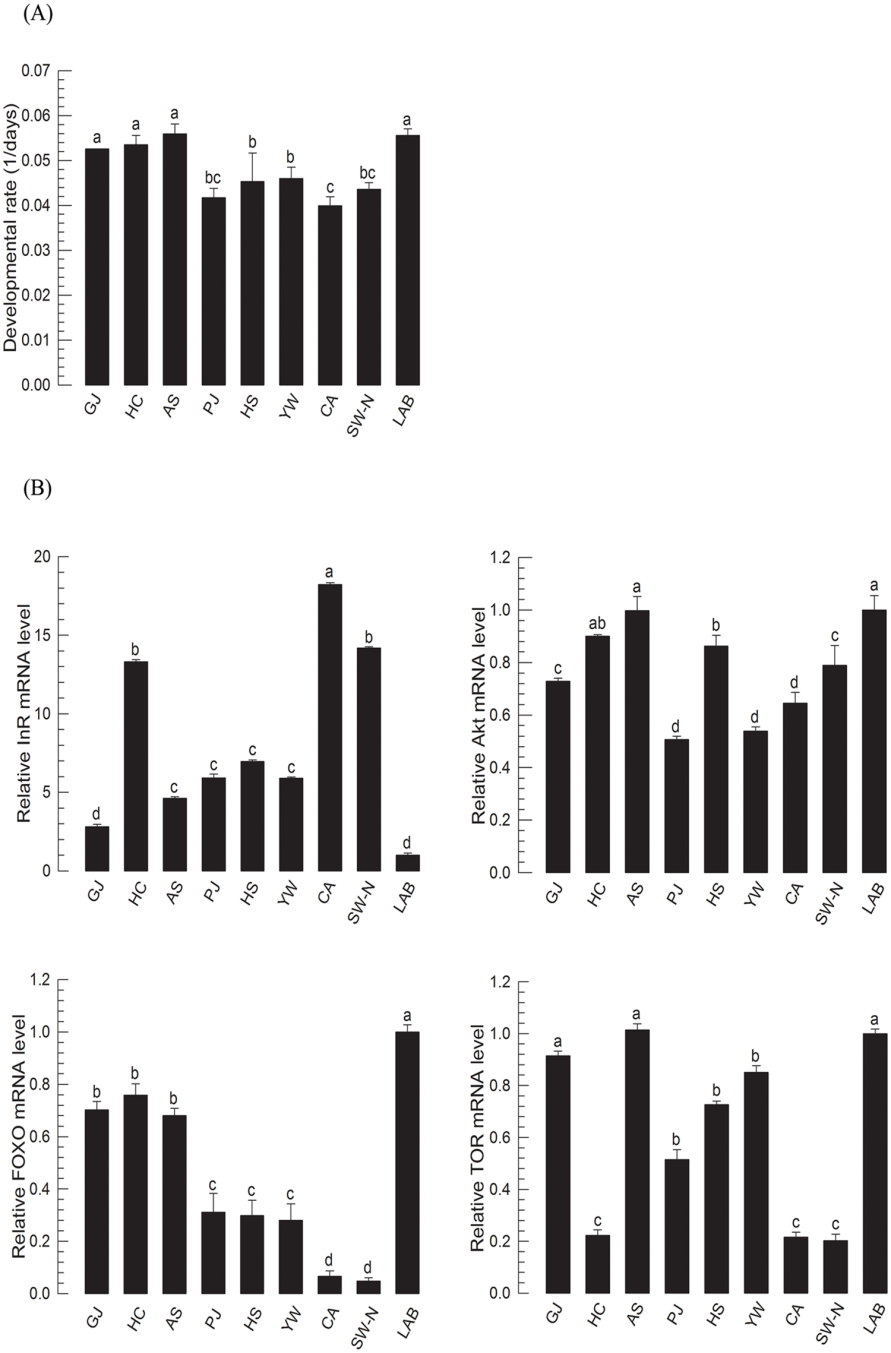
Effect of different genetic backgrounds on larval growth of *M*. *vitrata*. Growth patterns of *M*. *vitrata* larvae were obtained from a laboratory strain reared with artificial diet (‘LAB’) and eight local populations: Gyoungju (‘GJ’), Hongchun (‘HC’), Ansan (‘AS’), Paju (‘PJ’), Youngwol (‘YW’), Hoeungsung (‘HS’), Suwon (‘SW-N’), and Chonan (‘CA’). (A) Variation in larval growth of different local populations under the same rearing conditions at constant temperature 25°C with a standard artificial diet. Developmental rate was calculated by inverse of the total larval period (days). Each local population treatment used 30 larvae. (B) Change in expression levels of four IIS component genes (InR, Akt, FOXO, and TOR). These four genes’ expression levels in L5D1 larvae of eight local populations reared at constant temperature of 25°C and a standard artificial diet from L1 were quantified. Each treatment used nine randomly selected larvae. mRNA levels were relatively quantified and compared to mRNA level in L5D1 larvae reared in the laboratory with artificial diet. Actin was used as reference gene of RT-qPCR to normalize target gene’s expression level. Different letters above standard deviation bars indicate significant difference among means at Type I error = 0.05 (LSD test). (C) T-test between the expected larval period (L1D1-L5D5) from regressions obtained from IIS gene expression levels and the observed larval period (L1D1-L5D5). Asterisk indicates significant difference between expected and observed values. ‘NS’ represents no significance at Type I error = 0.05.

The IIS-growth rate model of *M*. *vitrata* was applied to natural populations under the same rearing temperature and diet conditions. Different local populations of *M*. *vitrata* were collected to obtain various genetic backgrounds and developmental rates (Fig 5). A total of 19 populations were compared in genetic backgrounds by RAPD analysis. There were three clusters: exotic populations (SW-O, SW-N, CHINA, MYA), domestic 1 population (LAB, AS, SS, HC, YP, YW, GH, IJ, IN), and domestic 2 population (PC, HS, TA, PJ, MY, YJ). However, these three clusters were independent of geographical locations (S4 Fig). For example, two proximal locations (PC and YW) were separated in genetic clustering, but two remote locations (GH and YW) were clustered. Eight field populations were successfully reared in the laboratory to obtain the next generation. Their following generations were reared at 25°C rearing temperature on artificial diet and their growth rate and IIS expression levels were compared to laboratory reared population (Fig 6). Under the same diet and rearing temperature, these field populations showed variations in developmental rates (Fig 6A). They also showed different expression levels of IIS components (Fig 6B). When four IIS-growth models were applied, 50% of field populations were well explained by one of these four models (Fig 6C).

## Discussion

In our previous study, we have annotated IIS components of *M*. *vitrata* from a larval transcriptome and analyzed their gene expression levels in all developmental stages from egg to adult [20]. IIS mediates various physiological processes such as growth, metabolism, immunity, life span, and reproduction [8]. The present study tested a physiological role of *M*. *vitrata* IIS in mediating larval growth.

IIS mediated the larval growth rate of *M*. *vitrata* because a vertebrate insulin injection stimulated the larval growth. Developmental rate was significantly increased after injecting 0.1 μg or higher doses of porcine insulin per larva. However, the increase of pupal size required higher injection doses (> 1 μg or higher per larva). *M*. *vitrata* has been predicted to have two ILPs, which are expressed in the brain and other tissues (unpublished data), such as fat body as seen in ILP genes of other lepidopteran species [26]. The stimulatory effect of the porcine insulin on larval development suggests that ILPs of *M*. *vitrata* might play a crucial role in larval development. This speculation was supported by RNAi of IIS components and subsequent developmental retardation. Four IIS components were analyzed by a loss-of-functional assay using RNAi approach. Mining genomes of various insects has revealed all components of mammalian IIS [27]. These four IIS components used in this current study were based on a previous study showing that they were expressed during larval stage with significance in controlling trehalose levels in the larval hemolymph [20].

InR is expressed in all larval tissues [20]. It has been regarded as the sole receptor for insect ILPs [9,28]. However, expression levels of InR may follow larval development as seen in *S*. *littoralis*, in which InR expression in the fat body was fluctuated according larval-pupal development [29]. Alteration of immature development by InR RNAi has been well demonstrated in the brown citrus aphid, *Aphis* (*Toxoptera*) *vitricidus* [30]. Two InRs (AcInR1 and AcInR2) are increased during nymph-adult transition and their RNAi by dsRNA feeding can result in a variety of malformed adult phenotypes along with significant nymphal mortality [30].

Akt (also known as protein kinase B) is one of pleckstrin homolog (PH)-domain-containing proteins that can bind to PIP3. It is activated by PDK or TOR [31]. Activated Akt phosphorylates S6 ribosomal kinase (S6K) which stimulates protein translation for cell growth [32]. Inhibition of Akt activity has been demonstrated in *Drosophila* Tribbles (Trbl) which is the founding member of Trbl family of kinase-like docking proteins that can modulate cell signaling during proliferation, migration, and growth [33]. Manipulation of *Trbl* expression modulates cell size because its direct binding to Akt prevents Akt phosphorylation of downstream IIS components such as FOXO and S6K [34]. Thus, RNAi of Akt that leads to retarded larval development and reduced pupal size of *M*. *vitrata* can be explained by preventing its downstream activation for cell growth and proliferation.

FOXO, a transcriptional activator, regulates cell fate, cell survival, cell differentiation, detoxification, and metabolism in the nucleus [35]. Akt phosphorylates and inhibits FOXO by preventing its entry into the nucleus in which FOXO activates transcription of up to 2,000 genes in *Drosophila* [36]. FOXO also regulates cAMP signaling by directly stimulating adenylate cyclase gene expression in the corpora allata, indicating that FOXO can integrate both cAMP signaling and IIS to adapt organismal growth to existing nutritional conditions [37]. In our current study, retarded development of *M*. *vitrata* induced by RNAi of FOXO can be explained by altered optimal growth conditions maintained by FOXO transcriptional activity. However, in cricket *Gryllus bimaculatus*, FOXO RNAi increased body size while RNAi against other IIS components led to reduced body sizes [38]. This contrasting result might be due to alteration of FOXO activity in prothoracic gland (PTG) involved in ecdysteroid biosynthesis. In *D*. *melanogaster*, RNAi of FOXO in PTG has resulted in failed metamorphic development and late developmental rate along with increased body size because FOXO directly interacts with Ultraspiracle (USP), part of the ecdysone receptor (EcR), during larva-to-pupal transition without interfering with EcR-USP heterodimer formation [39].

TOR pathway is activated by nutrient-sensing through amino acid transporters. It then activates S6K and 4E-BP which will activate 40S ribosomal protein S6 and eIF4E, respectively, to initiate protein translation [40]. One important growth-regulatory target of TOR signaling is ribosome biogenesis via RNA polymerase I transcription factor (TF_IA_). TOR inhibition can reduce TF_IA_ level, delay larval development, and decrease body growth of *D*. *melanogaster* [41]. Thus, suppression of TOR expression level in *M*. *vitrata* might have impaired larval development.

IIS-mediated larval growth was further supported by correlation assays under different environmental conditions such as ambient temperature and nutrient. With increasing ambient temperature, larval developmental time was decreased along with changes in expression levels of IIS components in 5^th^ instar first day (L5D1) larvae. Expression levels of Akt and TOR were increased with increasing ambient temperatures from 15°C to 25°C while those of InR and FOXO were decreased. As explained above, FOXO activity is negatively regulated by IIS induced under growth-favorable conditions. It is decreased at higher temperatures. Expression of InR is regulated by FOXO [42] whose activity is decreased with increasing ambient temperatures. In contrast, expression levels of Akt and TOR were positively correlated with ambient temperature between 15°C and 25°C, suggesting that they could behave like growth-facilitating signaling factors in IIS. When *B*. *mori* larvae resumed growth after starvation, they showed increased expression levels of growth-facilitating signaling factors in IIS in 2 h [43]. However, their expression levels were not increased at 30°C, although their larvae exhibited significant increase of growth rate. Thus, there might be some negative feedback controls of IIS component expression at high ambient temperatures. Rearing temperature experiments supported that IIS expression was associated with larval growth of *M*. *vitrata*. However, there was no linear relation between IIS gene expression and growth rate at different temperatures.

Nutrients can significant change larval development of *M*. *vitrata*. Developmental rates were dependent on diet quality. They were increased in an order from poor to good quality diets. Expression levels of four IIS components were quantified and compared to those in larvae reared on the standard diet. Except InR, expression levels of the other three IIS components exhibited positive correlations with larval growth rates. InR expression showed negative correlation with larval growth rate. This might be due to the fact that FOXO activates InR expression by its nuclear translocation under poor nutrient conditions [42]. However, the positive correlation of FOXO expression with growth rate suggests that its expression is dependent on IIS. Based on these correlations, four regression formulas were estimated and applied to growth patterns of larvae reared on six natural hosts at 25°C. Growth rates of more than half of test treatment groups were well predictable by these IIS-growth regression formulas. Interestingly, under the same temperature and nutrient conditions, natural populations of *M*. *vitrata* exhibited differential growth rates, suggesting different genetic backgrounds which were confirmed by RAPD polymorphism analysis. These IIS-growth regression formulas were also practicable to discriminate developmental rate variation with some deviations.

Regression formulas between IIS component expression and growth rate based on their functional correlation could be used to predict environmental effect on larval growth of *M*. *vitrata*. This kind of epigenetic control of growth pattern cannot be predicted by traditional day degree (DD) growth model which is based on larval growth at cumulative DD above low threshold temperature or development rate summation methods [44]. However, the application of IIS-growth model is limited to prediction of larval growth rate at a constant temperature of 25°C. To overcome this limitation, further correlation assays should be performed at various temperatures. To achieve optimal prediction, all IIS components and their interactions need to be considered. Several mathematical models have been developed to explain a network of IIS component activities and their interactions [45], in which a comprehensive mathematical model was devised as a qualitative model of insulin-TOR-MAPK network [46]. Thus, a novel insect growth model is likely to be developed by incorporating relative expression levels of IIS components proposed in this study.

## Supporting information

S1 Table Artificial diet compositions. (DOCX)

S2 Table Primers used for RT-qPCR in this study. (DOCX)

S1 Fig Domain structure and phylogenetic analysis of IIS signaling pathway genes (InR, Akt, FOXO, TOR). Significant domains of *M*. *vitrata* IIS pathway genes were identified by Pfam. Amino acid sequences of insulin receptor (InR) were retrieved from GenBank with accession numbers of NM_001043546.1 for *Bombyx mori*, FJ169464.1 for *Manduca sexta*, XM_021336792.1 for *Helicoverpa armigera*, MF443095.1 for *Maruca vitrata*, XM_011569614.1 for *Plutella xylostella*, XM_015979260.1 for *Tribolium castaneum*, XM_011527989.2 for *Homo sapiens*, BK008012.1 for *Apis mellifera*, and U18351.1 for *Drosophila melanogaster*. Amino acid sequences of protein kinase B (Akt) were retrieved from GenBank with accession numbers of ARS43574.1 for *H*. *armigera*, NP_005154.2 for *H*. *sapiens*, NP_033782.1 for *Mus musculus*, NP_732113.3 for *D*. *melanogaster*, ABY50539.1 for *B*. *mori*, AAP37655.1 for *Aedes aegypti*, NP_001023645.1 for *Caenorhabditis elegans*, XM_022971351.1 for *Spodoptera litura*, and MG657022 for *M*. *vitrata*. Amino acid sequences of Forkhead box protein O (FOXO) were retrieved from GenBank with accession numbers of AKQ99123.1 for *H*. *armigera*, NP_996204.1 for *D*. *melanogaster*, XP_011214722.1 for *Bactrocera dorsalis*, AFD99125.1 for *B*. *mori*, NP_002006.2 for *H*. *sapiens*, ABK76646.1 for *A*. *aegypti*, AEI86721.1 for *Culex pipiens*, NP_001251490.1 for *C*. *elegans*, NP_062714.1 for *M*. *musculus*, and MG657023 for *M*. *vitrata*. Amino acid sequences of target of rapamycin (TOR) (FOXO) were retrieved from GenBank with accession numbers of NP_004949.1 for *H*. *sapiens*, NP_524891.1 for *D*. *melanogaster*, XP_019844581.1 for *B*. *dorsalis*, NP_064393.2 for *M*. *musculus*, XP 011560435.1 for *P*. *xylostella*, NP 001171774.1 for *B*. *mori*, XP 013148240.1 for *Papilio polytes*, XP 022822531.1 for *S*. *litura*, XP 021189764.1 for *H*. *armigera*, and MG657024 for *M*. *vitrata*. Amino acid sequences were aligned with MEGA6. Numbers on nodes represent bootstrap values after 1,000 replications. (A) Domain and phylogenetic analysis of InR. RLD: receptor L domain; Furin: Furin-like cysteine region; PKT: Protein kinase catalytic domain. (B) Domain and phylogenetic analysis of Akt. PH: Pleckstrin homology domain; Pk: Protein kinase domain; PKC: Protein kinase C domain. (C) Domain and phylogenetic analysis of FOXO. PKC: Protein kinase C. (D) Domain and phylogenetic analysis of TOR.
(DOCX)

S2 Fig RNAi efficiencies of four IIS component genes. ‘dsCON’ represents dsRNA control using a viral gene CpBV302. dsRNA (1 μg) was injected to L5D1 larvae. Each qPCR measurement was replicated with three independent samples. Different letters above standard deviation bars indicate significant difference among means at Type I error = 0.05 (LSD test).
(DOCX)

S3 Fig Regression analyses of four IIS genes (InR, Akt, FOXO, TOR) between their expression levels and developmental rates at different rearing temperatures. Dot lines represent linear regression lines.
(DOCX)

S4 Fig A map indicating collection sites of different local populations of *M*. *vitrata* used in this study. Based on RAPD analysis, there are three clusters: domestic group 1 (blue color spots), domestic group 2 (gray color spots), and exotic group (red color spots). Local coordinates are described in Fig 5.
(DOCX)
