# Supplementary material for: Application of insulin signaling to predict insect growth rate in Maruca vitrata (Lepidoptera: Crambidae)
Source: PLoS One. 2018 Oct 4;13(10):e0204935. doi: 10.1371/journal.pone.0204935 (PMC6171882; doi:10.1371/journal.pone.0204935)
Supplement: S1 Table — (DOCX) [file pone.0204935.s001.docx]

**S1 Table**. **Artificial diet compositions**

| Components | Standard  diet (g) | Adzuki bean diets (g) | | | Cowpea diets (g) | | |
| --- | --- | --- | --- | --- | --- | --- | --- |
|  |  | T1 | T2 | T3 | T1 | T2 | T3 |
| Agar | 13.0 | 13.0 | 13.0 | 13.0 | 13.0 | 13.0 | 13.0 |
| Glucose | 10.0 | 10.0 | 10.0 | 10.0 | 10.0 | 10.0 | 10.0 |
| Vitamin | 5.0 | 5.0 | 5.0 | 5.0 | 5.0 | 5.0 | 5.0 |
| Ascorbic acid | 4.0 | 4.0 | 4.0 | 4.0 | 4.0 | 4.0 | 4.0 |
| Sorbic acid | 1.0 | 1.0 | 1.0 | 1.0 | 1.0 | 1.0 | 1.0 |
| Cellulose | 10.0 | 10.0 | 10.0 | 10.0 | 10.0 | 10.0 | 10.0 |
| Wheat germ | 10.0 | - | - | - | - | - | - |
| Adzuki bean | 20.0 | 105.0 | 50.0 | 20.0 | - | - | - |
| Soybean | 75.0 | - | - | - | - | - | - |
| Cowpea | - | - | - | - | 105.0 | 50.0 | 20.0 |
| Cholesterol | 3.0 | 3.0 | 3.0 | 3.0 | 3.0 | 3.0 | 3.0 |
| β-Sitosterol | 1.0 | 1.0 | 1.0 | 1.0 | 1.0 | 1.0 | 1.0 |
| Methyl-*p*-hydroxybenzoate | 1.5 | 1.5 | 1.5 | 1.5 | 1.5 | 1.5 | 1.5 |
| Aureomycin | 0.5 | 0.5 | 0.5 | 0.5 | 0.5 | 0.5 | 0.5 |
| Fumidil B | 0.4 | 0.4 | 0.4 | 0.4 | 0.4 | 0.4 | 0.4 |
| Water | 800.0 | 800.0 | 800.0 | 800.0 | 800.0 | 800.0 | 800.0 |
