# Supplementary material for: Application of insulin signaling to predict insect growth rate in Maruca vitrata (Lepidoptera: Crambidae)
Source: PLoS One. 2018 Oct 4;13(10):e0204935. doi: 10.1371/journal.pone.0204935 (PMC6171882; doi:10.1371/journal.pone.0204935)
Supplement: S2 Table — (DOCX) [file pone.0204935.s002.docx]

**S2 Table. Primers used for RT-qPCR in this study**

| Gene | Primer sequence (5' - 3') | Annealing temperature (°C) |
| --- | --- | --- |
| Insulin receptor (InR) | F: GTCGCAACAAGTACGACAGC | 58 |
|  | R: CAGGTAGGTCTTCAGGTCGC |  |
| Protein kinase B (Akt) | F: GAGATACTAACCGCAGCCTTTC | 55 |
|  | R: CACTTACGACACGTTCTCACTATC |  |
| Forkhead box protein O (FOXO) | F: AATCGTTCAGGTAGGCAATCC | 52 |
|  | R: CGCGCCCGAATCCTATAAAT |  |
| Target of Rapamycin (TOR) | F: GCTTTCTTTGGTCTTGAGGAAC | 55 |
|  | R: TCAGCACAACGTCGAACA |  |
| β-Actin | F: CATCACCATCGGAAACGAAAGG | 52 |
|  | R: ATACTGTGTTGGCGTACAGGTC |  |
