# Supplementary figures and images for: Application of insulin signaling to predict insect growth rate in Maruca vitrata (Lepidoptera: Crambidae)
Source: PLoS One. 2018 Oct 4;13(10):e0204935. doi: 10.1371/journal.pone.0204935 (PMC6171882; doi:10.1371/journal.pone.0204935)

**(A)**

**
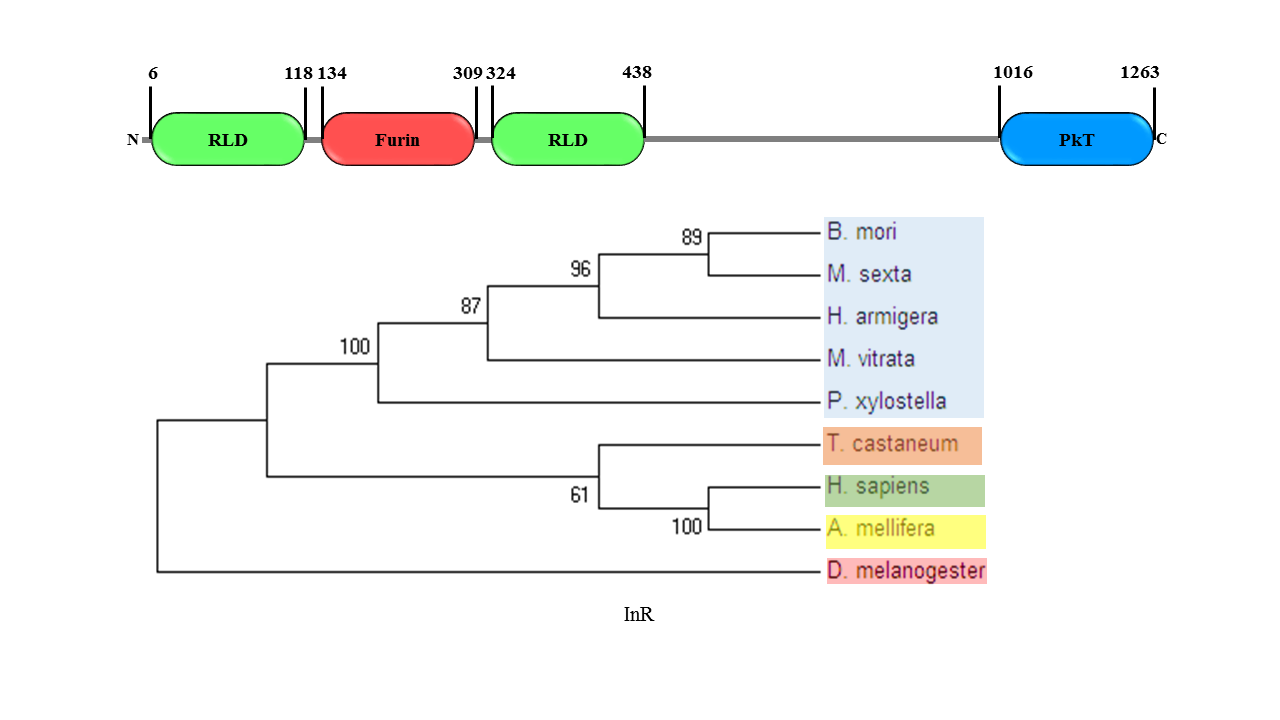
**

**(B)**

**
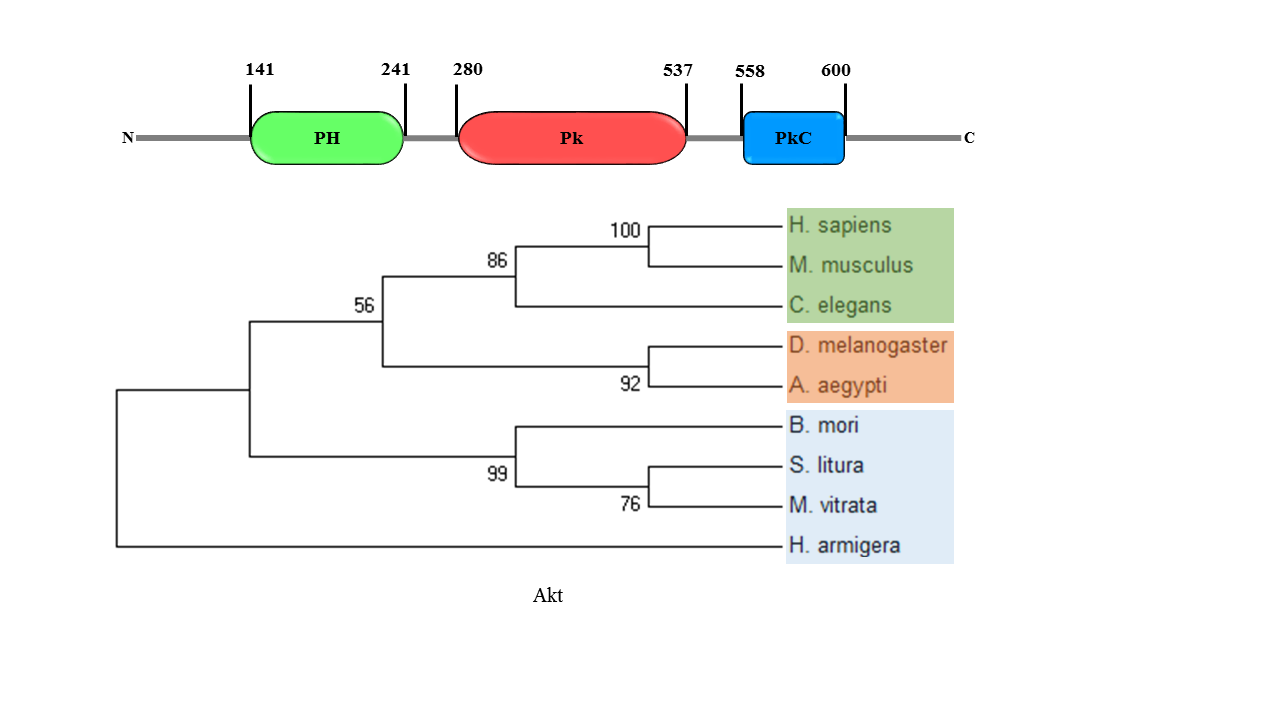
**

**(C)**

**
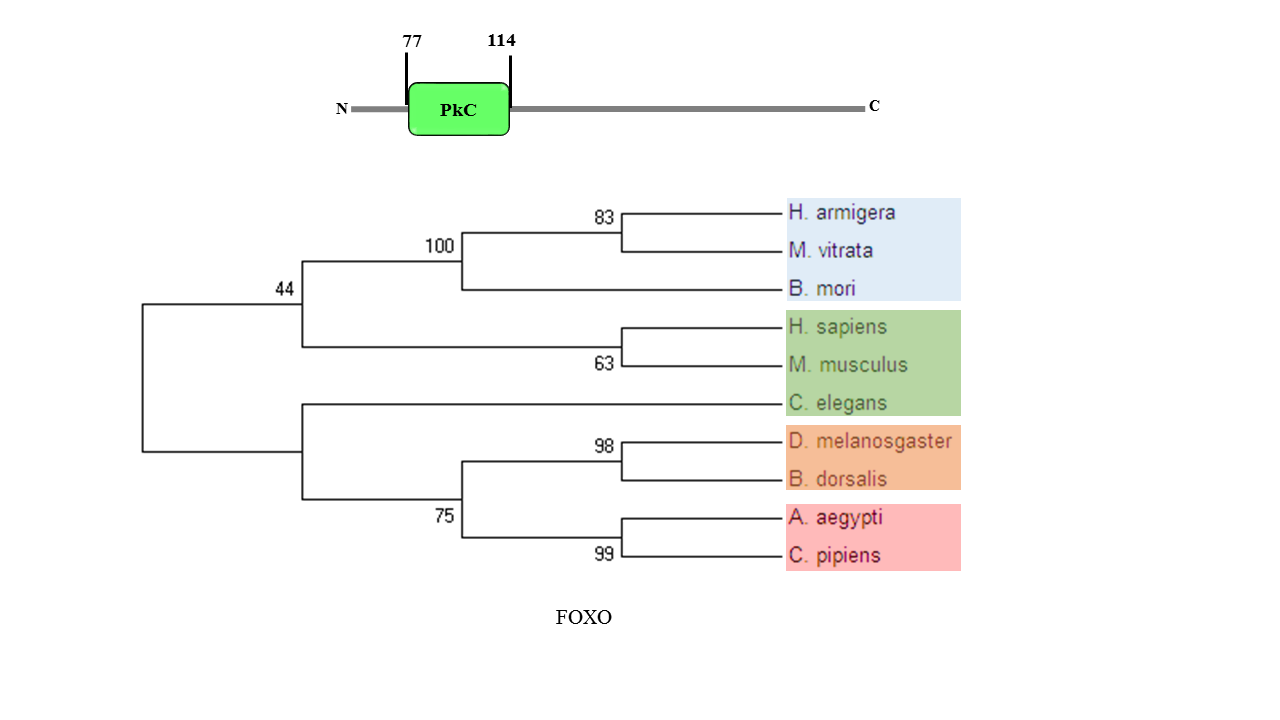
**

**(D)**

**
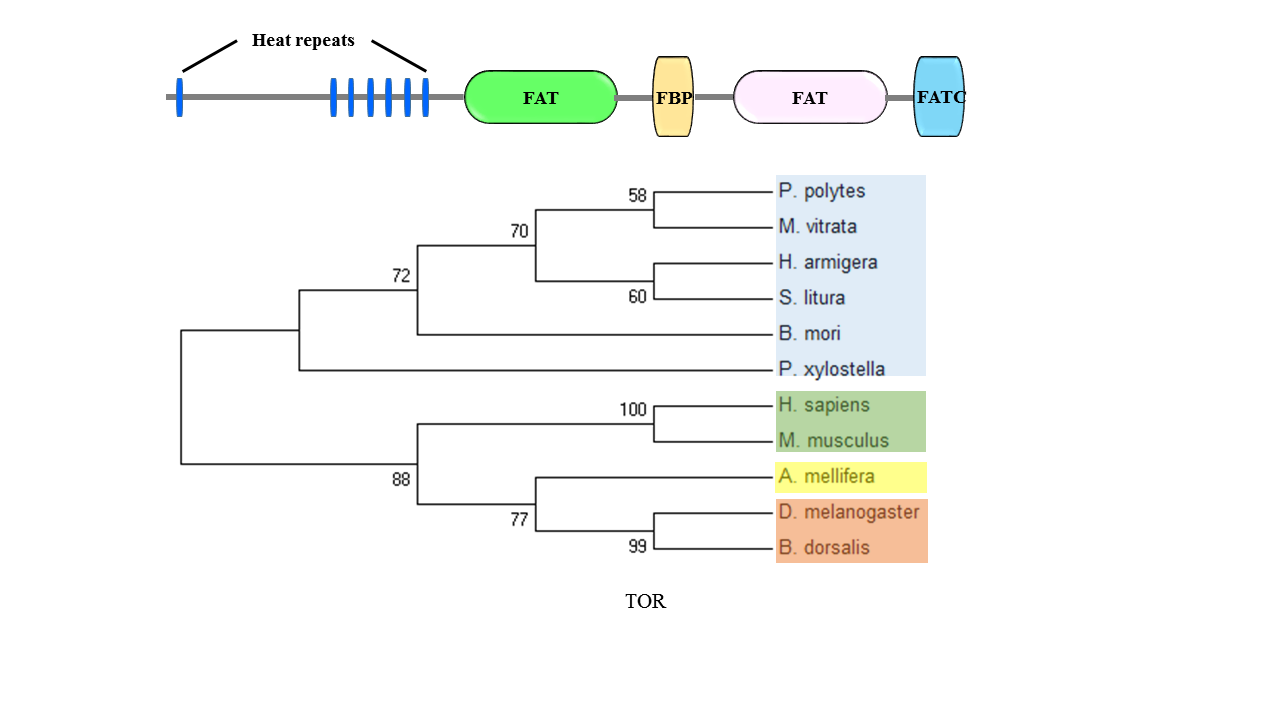
**

Supplement: S1 Fig — Significant domains of M. vitrata IIS pathway genes were identified by Pfam. Amino acid sequences of insulin receptor (InR) were retrieved from GenBank with accession numbers of NM_001043546.1 for Bombyx mori, FJ169464.1 for Manduca sexta, XM_021336792.1 for Helicoverpa armigera, MF443095.1 for Maruca vitrata, XM_011569614.1 for Plutella xylostella, XM_015979260.1 for Tribolium castaneum, XM_011527989.2 for Homo sapiens, BK008012.1 for Apis mellifera, and U18351.1 for Drosophila melanogaster. Amino acid sequences of protein kinase B (Akt) were retrieved from GenBank with accession numbers of ARS43574.1 for H. armigera, NP_005154.2 for H. sapiens, NP_033782.1 for Mus musculus, NP_732113.3 for D. melanogaster, ABY50539.1 for B. mori, AAP37655.1 for Aedes aegypti, NP_001023645.1 for Caenorhabditis elegans, XM_022971351.1 for Spodoptera litura, and MG657022 for M. vitrata. Amino acid sequences of Forkhead box protein O (FOXO) were retrieved from GenBank with accession numbers of AKQ99123.1 for H. armigera, NP_996204.1 for D. melanogaster, XP_011214722.1 for Bactrocera dorsalis, AFD99125.1 for B. mori, NP_002006.2 for H. sapiens, ABK76646.1 for A. aegypti, AEI86721.1 for Culex pipiens, NP_001251490.1 for C. elegans, NP_062714.1 for M. musculus, and MG657023 for M. vitrata. Amino acid sequences of target of rapamycin (TOR) (FOXO) were retrieved from GenBank with accession numbers of NP_004949.1 for H. sapiens, NP_524891.1 for D. melanogaster, XP_019844581.1 for B. dorsalis, NP_064393.2 for M. musculus, XP 011560435.1 for P. xylostella, NP 001171774.1 for B. mori, XP 013148240.1 for Papilio polytes, XP 022822531.1 for S. litura, XP 021189764.1 for H. armigera, and MG657024 for M. vitrata. Amino acid sequences were aligned with MEGA6. Numbers on nodes represent bootstrap values after 1,000 replications. (A) Domain and phylogenetic analysis of InR. RLD: receptor L domain; Furin: Furin-like cysteine region; PKT: Protein kinase catalytic domain. (B) Domain and phylogenetic ana [file pone.0204935.s003.docx]

**
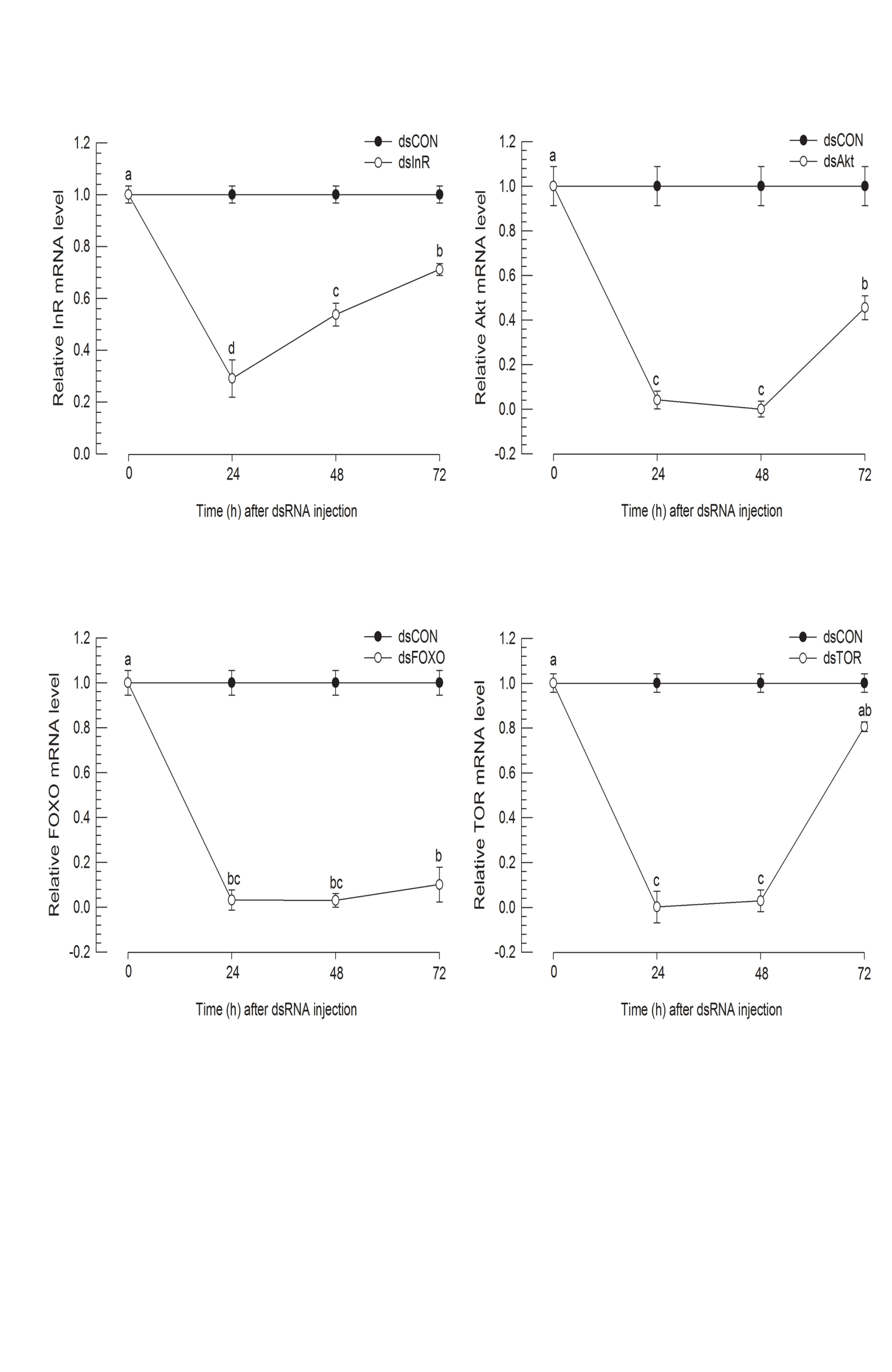
**

Supplement: S2 Fig — ‘dsCON’ represents dsRNA control using a viral gene CpBV302. dsRNA (1 μg) was injected to L5D1 larvae. Each qPCR measurement was replicated with three independent samples. Different letters above standard deviation bars indicate significant difference among means at Type I error = 0.05 (LSD test). (DOCX) [file pone.0204935.s004.docx]

**
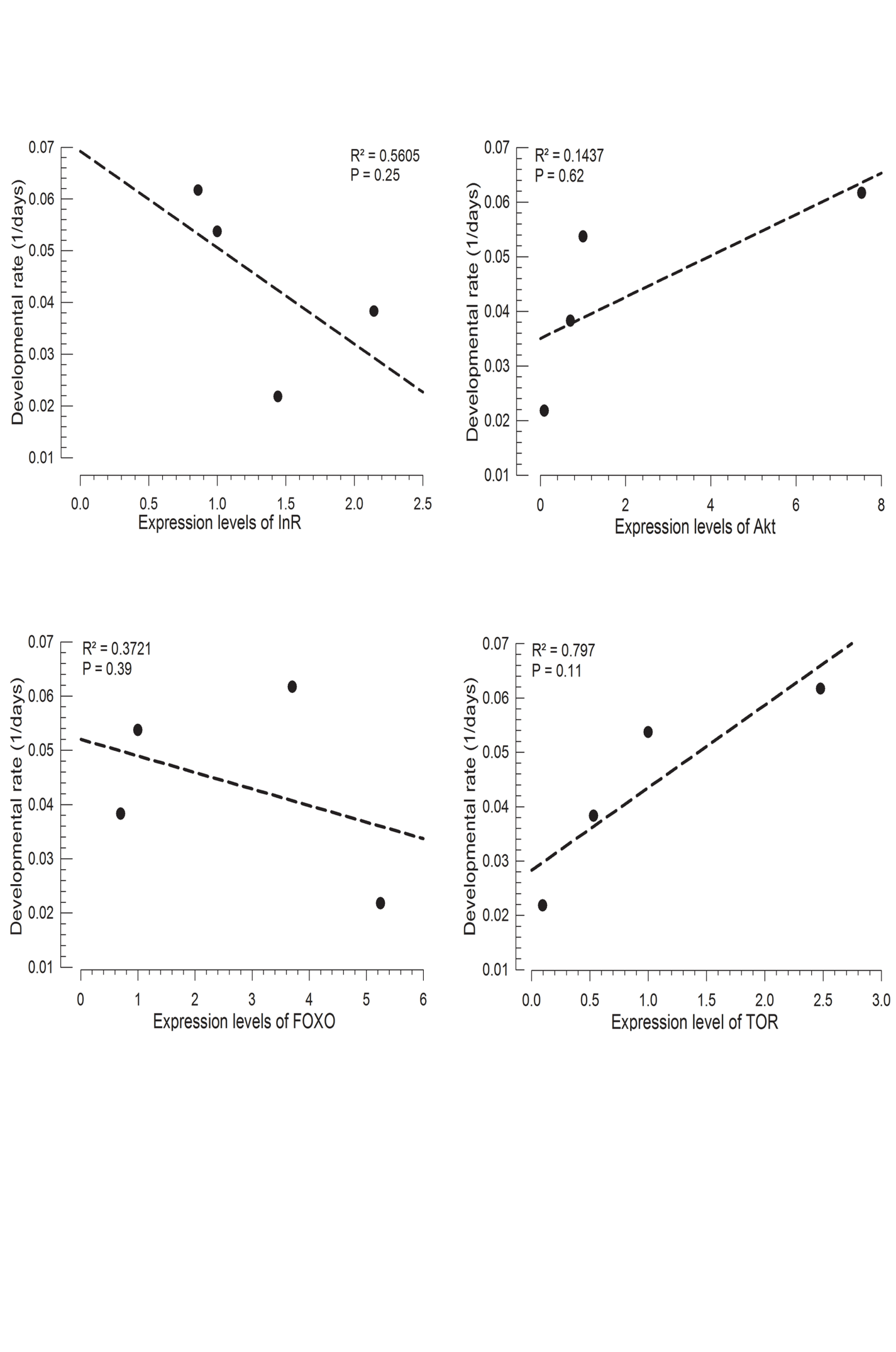
**

Supplement: S3 Fig — Dot lines represent linear regression lines. (DOCX) [file pone.0204935.s005.docx]

**
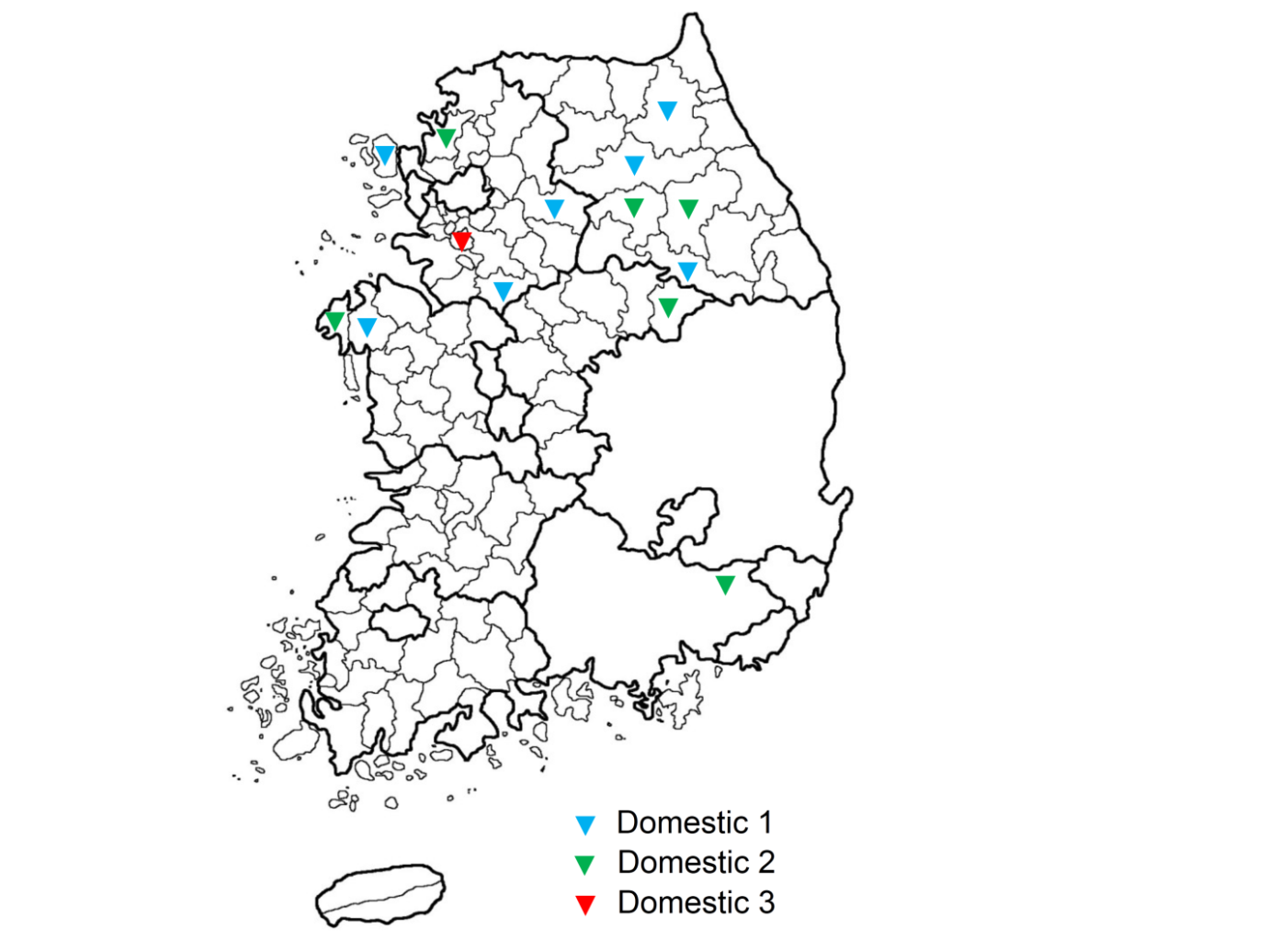
**

Supplement: S4 Fig — Based on RAPD analysis, there are three clusters: domestic group 1 (blue color spots), domestic group 2 (gray color spots), and exotic group (red color spots). Local coordinates are described in Fig 5. (DOCX) [file pone.0204935.s006.docx]
